# In enemy hands: the Byzantine experience of captivity between the seventh and tenth centuries

**DOI:** 10.1111/emed.12642

**Published:** 2023-06-22

**Authors:** Grigori Simeonov

**Affiliations:** ^1^ Institute for Byzantine and Modern Greek Studies Vienna

## Abstract

The present paper deals with forced migration experienced by subjects of the Byzantine Empire captured by foreign enemies in the context of warfare between the seventh and the tenth centuries. The focus of the first part is on the scenarios faced by individuals and groups when an enemy had taken control of a settlement or a larger territory. The second part discusses aspects of the role social status and gender played in the process of being taken over and then (possibly but not necessarily) held in captivity. Although one can trace similarities in the way captors treated their captives on different occasions, an overgeneralizing approach can prove misleading, distracting us from the dynamics of the consequences that war and abduction had on both the agency of the victor and the fate of the loser in the early Middle Ages.

## Introduction

Isauria, ad 650, 5,000 people; Reggio di Calabria, ad 901, 17,000 people; Thessaloniki, ad 904, 22,000 people; Tiriolo in Calabria, ad 929/30, 12,000 people. 1 These are just some examples of the numbers we possess relating to Byzantines who were taken captive during military campaigns starting from the year 600. Even if one has doubts about the accuracy of these numbers – though some are preserved in accounts of eyewitnesses such as John Kaminiates – there can be little doubt about the role that armed conflicts have long played in bringing about forced migration in the human past. 2 If for a moment we set aside ideology, the objective of warfare has revolved around conquering – or conversely, protecting – land, people, and/or materials (in the form of valuables, resources, or specific goods). Drawing a sharp distinction between these categories might be difficult if not misleading, because an armed conflict could have as justification a combination of several if not all of these motives. In terms of migration and mobility and their impact on the life of medieval people, the conquest of a territory could result in the flight of the local population and the resettlement of both old and new residents. When the targets of seizure were humans and valuables, the consequence for the losers was abduction and captivity that caused a breaking (sometimes temporary but often definitive) of the bond between people and their homeland. 3

In what follows, I will try to offer a brief glimpse of the various experiences of captivity that subjects of the Byzantine Empire were forced to face during the early Middle Ages. 4 In line with the theme of this special issue, this article focuses specifically on forced migration out of Byzantine territory and the personal agency of the enemy on the fate of the captives. 5 In addition, this study aims to shed some light on the role that the social background of Byzantine captives played in the process of being taken and then held in captivity. 6 By abducting hundreds, sometimes even thousands, of combatants and civilians, the enemy pursued practical as well as ideological considerations. On the one hand, opponents damaged the military, demographic, economic, and financial power of their rivals and optimized their own. 7 On the other, captives, especially those of high status as well as prisoners of war, could be instrumentalized as symbols of superiority over the defeated enemy, both in the captor’s country and abroad. They could also be used as bargaining tools during diplomatic negotiations to secure advantageous treaties. 8

## Setting the parameters

Some initial remarks on terminology, the spatial and chronological framework of this study, and the main sources would seem in order before we turn our attention to the fate of Byzantine captives. Modern scholarship on forced migration distinguishes between combatants (prisoners of war) and civilians (captives). 9 The first fell into the arms of the enemy on the battlefield or in a sacked settlement, whereas the latter were usually victims of raids or sieges. However, ‘war captives’ or ‘hostages’ are used as general terms for both combatants and civilians who were subjugated and subsequently abducted. 10 Moreover, life in captivity was a fluctuating and shifting state, which sometimes makes drawing a rigid boundary between its various forms a difficult task. Medieval Greek sources, in any case, tend to use the same designation (*aichmalotoi*), making little distinction between combatants and civilians taken into captivity. 11

The Arab conquest in the east, and the Avar, Slavic and Bulgar incursions in the Balkans, each represented a significant challenge to Byzantium, marked by a series of military conflicts of varying intensity and scale. 12 Although the empire ‘would not die’ and succeeded in pushing the Arabs back and securing Asia Minor, 13 the ninth century witnessed the loss of three major islands (Crete, Cyprus, and Sicily), subjecting Byzantium to a constant threat of pillaging and depredation from Arab fleets in southern Italy, Dalmatia, and the Aegean. 14 These conflicts – it matters not whether they were official acts between two sovereign authorities or raids by pirates and brigands – went hand in hand with death, destruction, resettlement, and life in captivity for thousands of imperial subjects. 15

Three major military blows to Byzantium during the period under consideration resulted in forced mobility that is relatively well documented in medieval sources. These were the fall of Amorion in 838, the capture of Syracuse in 878, and the sack of Thessaloniki in 904. 16 Byzantine historical texts make mention of them, but the tendency is to compress the fate of the victims into an anonymous mass, thus foregrounding general event over individual experience. For two of these occasions we possess eyewitness accounts from residents who were taken into captivity by the conqueror. 17 These are the works of Theodosios the Monk on the fall of Syracuse 18 and of John Kaminiates on the sack of Thessaloniki, both of them labelled by their authors *epistolai*, ‘letters’. 19 Although the circumstances under which both texts emerged as well as the place and time they were written are still a matter of scholarly discussion, it is possible that at least their draft versions go back to the period when the authors lived in Arab captivity. 20 This makes them unique sources of information, though there are still some idiosyncrasies to which the reader interested in the way Byzantines experienced the debacles of war and captivity must pay attention. 21 Since Theodosios and John were members of the local elite, this is reflected in both their fate as captives and the nature of their narratives. In the ego‐accounts of their abductions, the emphasis is on the way high‐status captives experienced conquest and depredation. 22 However, although higher social status could often provide better chances of surviving the pillaging of the conquest and of arriving in the captor’s homeland without being sold into slavery during the transfer, it was never a guarantee of survival and ultimate return. As will be explored further below, because taking captives was intrinsic to armed conflicts and because warfare has always had its own rules, the fate of captives was subordinated to a great many factors and circumstances that could rapidly change within a short period of time and therefore affect the captives’ lives dramatically. 23

As for the third major Byzantine loss mentioned above, the sack of Amorion, in addition to the detailed account of the Arab historian al‐Ṭabarī, the fall of the city and the fate of forty of its abducted defenders feature in one of the most famous works of Byzantine hagiography, the *Life of the 40 Martyrs of Amorion*. 24 Beyond the usual questions about topoi (commonplaces) that go hand in hand with the analysis of hagiographical works as a source for reconstructing historical events, 25 the historicity of the martyrs’ slaughter by the Arabs has recently been cast into question. 26 Yet, as Youval Rotman remarks, abduction and slavery were a common theme in Byzantine hagiography of the ninth and tenth centuries, and appear in numerous saints’ *Lives* as a cause for supernatural intervention. 27 This indicates that the threat of being taken captive during the period under consideration was only too real, thus making the motif of forced migration a familiar setting in terms of background for presenting a narrative to its audience. 28 Highly relevant here are the ideological conflicts between Christianity and Islam in the east and the south, and also between Christianity and paganism in the north. 29 These provided the incentive for Byzantine hagiography to portray saints (especially those who were active in territories where enemy assaults were frequent) as champions of Christianity to whom the believers – captives and/or their relatives – were to address their prayers and hopes for redemption. 30 Moreover, stories about captives who preferred death to rejecting Christ had to fulfil the needs of the important mission of hagiography in order to strengthen the faith of the emperor’s subjects, many of whom were exposed to the danger of abduction and thus could easily choose conversion to Islam over death in the name of Christ. 31

## Scenarios

### Death and humiliation

The start of the enemy’s assault usually meant the beginning of a series of calamities for the attacked, especially in cases of siege. 32 Death by sword or life in captivity were often preceded by hunger, exhaustion, emotional pressure, and pestilence. 33 If defeat ensued, there followed days or even weeks during which those whose lives had been spared for the moment awaited their transfer into the unknown. 34 Eye‐witnesses Kaminiates and Theodosios the Monk both tell of the terrible conditions of imprisonment in a captured city (Thessaloniki and Syracuse respectively), 35 in transport ships, 36 and at the final destination (Tarsos and Palermo). 37 The harsh conditions under which the captives from Amorion lived for seven years seem to have been no different. 38

Often those who had not died on the battlefield had to rely for their survival on the mercy – and sometimes even the whim – of the enemy commanders. 39 Since too many captives had fallen into his hands after the sack of Amorion in 838, Caliph al‐Muʽtaṣim (r. 833–42) ordered the killing of 4,000 men. 40 Moreover, the caliph could not afford to stay long in enemy territory. On the way back, the army crossed an area of desert, which was a formidable challenge for both army and captives. Consequently, al‐Muʽtaṣim ordered those who were not able to keep up with the marching troops – according to al‐Ṭabarī, about 6,000 people – to be slaughtered. Some of the captive Byzantines, however, managed to kill their captors and make their escape. 41 In the 950s, the Ḥamdānid emir of Aleppo Sayf al‐Dawla (r. 944–67) – a fervent rival of a series of Byzantine emperors – was defeated by the Byzantines and forced to flee. 42 Desperate to save his own life and those of his soldiers, he ordered the Byzantine prisoners of war to be killed. One of the few who succeeded in escaping was Niketas Chalkoutzes (*PmbZ* #25778). 43

Senior commanders theoretically had better chance of surviving because they could later be used in exchanges of prisoners of high social status. However, sources sometimes paint a picture that deviates from this principle. Arab historians narrate how on the way back from a pilgrimage to Medina, the Umayyad caliph Sulaymān ibn ʽAbd al‐Malik (r. 715–17) ordered some four hundred Byzantine prisoners to be killed along with their commander. 44 On the other hand, Aetios (*PmbZ* #108), the commander‐in‐chief of Amorion’s defenders, was captured but not killed, at least not right after the fall of the city (see above). 45 The high military officials who led the defence of Syracuse forty years later had a different fate after the Arabs breached the walls. The patrician, whose name Theodosios the Monk does not mention, was captured and killed eight days later, together with seventy of his soldiers. 46 One can speculate that the Arabs, who – according to the words of the Byzantine author – were impressed by his stoicism, preferred to kill such a prominent general rather than take him into captivity because of the long duration of the siege that had cost them nine months. 47

How insecure was the fate of even high‐status captives is shown in the case of the eunuch and *koubikoularios* Rhodophyles (*PmbZ* #26828). The Byzantine government had sent him to Sicily with a large quantity of gold, but illness prevented him from continuing his mission and he was forced to remain in Thessaloniki. Yet he managed to smuggle the gold outside of the city and sent it to the *strategos* of the Strymon theme. After the sack of Thessaloniki, he was brought to the Arab commander, Leo of Tripoli, who, not unexpectedly, showed great interest in establishing the whereabouts of the gold. Because Rhodophyles dared to be too direct in the negotiations, Leo grew angry, abused him verbally and ordered him to be beaten. The punishment was carried out with such severity that Rhodophyles did not survive. 48

Byzantine sources, and especially hagiographical works, present the life stories of prisoners of war or other captives – often zealous Christians – who did not hesitate in the face of danger and showed high morale and even a determination to die, preferring death over escape or imprisonment. By narrating these tales, Byzantine authors were trying to encourage their countrymen, and especially those engaged in fighting, to show determination in the face of the enemy. St Cosmas, for example, was kidnapped by Arab pirates on the shores of Crete but accepted his destiny at the sight of the approaching enemy, did not run away and thus did nothing unmanly or ignoble (*οὐκ ἄνανδρον, οὐκ ἀγεννὲς ἐνεδειξάμην οὐδέν, οὐ νῶτα στρέψαι, οὐ δραπετεῦσαι ἠθέλησα*). 49 The soldiers who are said to have protected Larissa against Arabs or Bulgarians in the early tenth century preferred to leave the besieged town to die in the mountains, rather than surrender. 50

Since courage plays a crucial role in warfare, humiliating the enemy by maltreating his captured soldiers who served as a symbolic representation for a greater whole was considered an effective means of lowering the morale of the rival and a manifesting a conquering power. 51 Thus medieval sources did not lack stories of the display of imperial military misfortune via the mutilation of Byzantine prisoners of war. After his victory over the troops of Leo VI (r. 886–912) in 894, the Bulgarian ruler ordered the imperial Khazar guardsmen to be sent back to their master, their noses cut off. 52 The Arabs were no less inclined. In 930, the *domestikos* Melias (*PmbZ* #25488) reached Arsamosata but was defeated by Naǧm, who was in charge of protecting the border. Four hundred Byzantine prisoners of war, including ten generals, were sent to the caliph in Baghdad, where they were paraded on camels. 53 Kaminiates and the other captives from Thessaloniki were forced to experience the same humiliation on their entry into Tripoli, where they were paraded under the shouts of the mocking crowd. 54

In some instances where the empire’s enemies displayed particular cruelty towards Byzantine captives, we may speculate that the motivation was revenge for previous actions by the imperial government or its officials. Thus, both Syrian and Greek authors present the sack of Amorion as a reaction to Theophilos’ campaign against the hometown of the caliph the year before and the devastation of the regions of Melitene and Samosata. 55 Furthermore, Byzantine historians describe a massacre of Bulgarian civilians committed during Nikephoros I’s campaign of 811. 56 We can suppose that the attempt to assassinate Bulgaria’s khan during the summit with Emperor Leo V (r. 813–20) in July 813 contributed to the harsh treatment of Byzantine prisoners and the forced relocation of the population of Thrace to regions north of the Danube, regardless of whether they were already living under Bulgarian control or were taken captive from imperial territories. 57 As for Niketas of Tarsos, the courageous defender of Syracuse, Theodosios the Monk says that his death was a penalty not only for his resistance to the Arabs but also for his continual curses against Muḥammad during the long siege. The punishment for this blasphemy against the prophet of Islam was one of the most gruesome ones recorded in Byzantine sources – Niketas was put on the ground before the Christian inhabitants, and was then skinned and dismembered. 58

### Ransom

Certain individuals, mainly those of high social status, tried to make arrangements for their safety before the enemy took them captive. This was possible not only because they possessed the necessary resources but also because they had better access to information as to how the fighting was going. Kaminiates’ family is a good example: they had taken the precaution of storing their valuables in a place known only to themselves, and after the fall of the city they gave the money to their Arab captors as a guarantee of their safety. 59 Later, Leo of Tripoli himself explained to them that this was one of the reasons why they had nothing to fear with regard to their lives; the other was that they were captured without offering his soldiers any resistance. 60 However, Kaminiates’ account makes clear that the Arabs meticulously checked whether all who said they had enough money to buy off their lives actually were so endowed. If it were not the case or the amount was insufficient, they were summarily put to death. 61 Here one has to make the distinction between paying a ransom in order to avoid death and then being taken captive, and paying a ransom to bring captivity to an end with the purpose of achieving deliverance. As for military commanders and their behaviour in the wake of seizure, we know that one of the officers in charge of the most damaged sector of Amorion’s defences in 838 (probably Boiditzes, *PmbZ* #1019) contacted the caliph and asked for a guarantee of safe conduct for himself and his family in return for helping the Arabs to take over the city. 62

The church’s role in Byzantine society also extended into these matters. Endeavouring to fulfil its mission of loving one’s neighbour and pleasing God by carrying out works of mercy in accordance with Christ’s own words (Luke IV.18), 63 it shared the notion with the other Abrahamic religions that one of the main manifestations of charity was to rescue captives. 64 It comes, therefore, as no surprise that medieval texts attest cases in which clergymen of various ranks committed to paying the ransoms for Byzantine captives. The church played an important role in raising alms for captured Christians, not only as an expression of solidarity towards a suffering brother in need but also because its own officials were often among the victims of abduction. After a summer campaign in 901, for example, Nizār ibn Muḥammad returned to Tarsos with 160 non‐Muslims, including abbots and deacons, as well as crosses and banners. The entire taking was then sent on to Baghdad. 65

In order to strengthen the religious solidarity among clergy and their flock, medieval Greek hagiography set the lives of some saints up as an example of Christian charity. Neilos of Rossano, the abbot of the Monastery of St Adrian near Rossano in Calabria, sold grain, wine, and other products that belonged to the monastery in order to raise ransoms for three monks who had been taken captive by the Arabs and were abducted to Sicily. 66 The *Life* of the same saint tells the story of the metropolitan Blatton, who sailed to Africa and returned to Calabria with many captives for whom he had paid ransoms. 67 The protagonists of these hagiographical stories about bishops or abbots, who spared neither time nor means in saving their Christian brothers taken into captivity, had parallels in real life. One of them was Malacenus, abbot and *episcopus sede sancti Basilii in regione Iberorum*. 68 Two Latin letters (*epistola encyclica*) written at the beginning of the tenth century – one by Patriarch Elias III of Jerusalem, the other by Pope Benedict IV – report the challenges Malacenus faced while himself a captive and at the same time his determination to raise ransoms for his brethren. His monastery was sacked by Turks who demanded twenty of the monks defame the Holy Cross. On their refusal, they were killed. The Turks abducted the other thirty brethren and the abbot Malacenus. Thanks to the help of some Christians who paid 120 *nomismata* in ransom, Malacenus and one of the monks were set free. However, the clergyman did not forget about his monks and sought donations first in Jerusalem and then in Rome. We do not know whether the patriarch and the pope supported him financially – Elias writes explicitly that he did not have enough means at his disposal – but the two high officials called on their flocks to devote their money and assist the bishop who was travelling ‘from city to city’ (*de civitate in civitatem*). 69 At approximately the same time, in the early 910s, a similar plea reached another high‐ranking religious official. Manso (or his son Mastalus I), prefect of Amalfi, approached his spiritual father Nicholas I Mystikos, patriarch of Constantinople, asking him for help in obtaining the ransom for Amalfitan prisoners who had been taken captive by the Arabs. The patriarch agreed to his request and sent a pound of gold to Amalfi. 70

Often it was families or kin who were expected to take responsibility and buy off their relatives. Kaminiates tells that before the Arab fleet sailed away, citizens of Thessaloniki who had managed to flee the city before its fall came back every day and tried to arrange ransoms for their relatives. 71 How ransoming happened on a micro level is very difficult to reconstruct due to the evidence being rare, fragmentary, or scattered: the sources usually focus on details concerning the fate of high‐status captives. 72 When we go down the social ladder, even hagiography gives us little help. As for accounts in charters or tax registers, the earliest data preserved dates back to the late tenth century. It is in the archives of Athonite and south Italian monasteries or in inscriptions that one comes across surnames like Aichmalotos, indicating former captivity. 73 Among the documents of the Iveron monastery one finds information as to how family members secured the means for their relatives’ ransoms. Thus, a document dating back to 1012 provides evidence for the sale of a piece of arable land by a widow Kalida. She sold it to Euthymios, abbot of the Lavra of St Clement, for 15 *nomismata* in order to pay the ransom for her son Basil, who was held in Arab captivity. 74

In the event of capture, the top military commanders could rely on the government or – on account of their social status – wealthy relatives to ransom them. 75 Thus, Abesalom Tzangoboutos and Seon Palatinos, two Byzantine generals who took part in the campaign against Samosata around 860, were captured by the Paulician ally of the Arabs, Karbeas. 76 Theophanes Continuatus says that they ‘gave sufficient money to Karbeas, having sent messages home requesting their ransom’. 77 In 998, Damian Dalassenos (*PmbZ* #21379), *doux* of Antioch, marched together with his troops against the Syrian city of Apameia and besieged it. Although the Byzantines enjoyed initial success, the Fāṭimid army arriving from Damascus to offer relief defeated the invaders. The *doux* was killed by a Kurdish soldier and his two sons Constantine and Theophylact were taken captive. They were brought to Cairo and stayed there for ten years before they were finally ransomed. 78

Sometimes, especially in cases of serious losses, it was the emperor who had to intervene and use his power to rescue captives of high status. One can speculate that it was thanks to the request of Empress Irene (r. 797–802) that Hārūn al‐Rashīd (r. 786–809) released her close associate Staurakios (*PmbZ* #6880). The caliph organized a major campaign in Asia Minor in 782 and with his troops penetrated as far as Chrysopolis. Staurakios had to negotiate with the enemy but, in neglecting his own security, was taken captive, thus making intervention from the highest state level necessary. 79 When he learned that Amorion, one of the leading urban centres of Asia Minor and hometown of the ruling dynasty, had been sacked by the Arabs in 838, one of the first things Emperor Theophilos (r. 829–42) did was to start negotiations with the victorious al‐Muʽtaṣim and try to arrange ransoming back the captives. In the circumstances, his initial attempt proved to be in vain. 80 What made the emperor’s intervention even more pressing was the fact that among the high‐ranking prisoners of war were some of his own relatives. 81 One needs only to mention Constantine Baboutzikos (*PmbZ* # 3932), magister and patrician, married to Sophia, sister of Empress Theodora, and thus the emperor’s brother‐in‐law. 82

However, Theophilos was not the last emperor said to have offered princely assistance in paying the ransom for high‐ranking Byzantine prisoners of war. In 964, Nikephoros II Phokas (r. 963–9) sent his navy against Sicily. Despite initial success, the army led by the patrician Niketas (*PmbZ* #25784) was defeated while trying to cross the Straits of Messina in what Arab sources call the Battle of the Straits. Manuel Phokas (*PmbZ* #24884), the emperor’s nephew in charge of the cavalry, was killed. However, Niketas, a eunuch and high court official, survived but was taken captive and sent to the Fāṭimid caliph of Egypt, al‐Muʽizz (r. 953–75). It fell to Nikephoros II to redeem Niketas and he applied various strategies to secure the liberation of his unfortunate general. Leo the Deacon says that the emperor promised to give the caliph the sword of Muḥammad in exchange for Niketas, and on pain of refusal even threatened the Arabs with war. 83 Be that as it may, it seems that the solution for Niketas’ deliverance was a substantial ransom, the rumours of which astonished even the western envoy, Liutprand of Cremona, during his stay in Constantinople. 84

### Sale into slavery

After the sack of Amorion, the victorious Caliph al‐Muʽtaṣim gave the order for the captives to be separated as follows – a third of them were reserved for the officers, a third for the Turks, while those remaining were to be sold into slavery. 85 His soldiers crowded the camp with captured men, women, and children. Then the captors started selling their human booty and also the goods they had looted. The whole procedure was completed within five days. 86 Athina Kolia‐Dermitzaki supposes that such actions allowed the captors to reduce the expense of transportation. 87 On one of the stops on the way to Tarsos, the fleet of Leo of Tripoli was visited by men who showed particular interest in some of his female captives. These were probably slave dealers shadowing the journey of the Arabs (on which, see further below).

Thus, another chapter of forced mobility of Byzantine captives was being written, though life as a Christian slave may have had as many highs and lows as that of prisoners awaiting their exchange. 88 Byzantine slavery is the topic of a meticulous study by Rotman, and I will therefore limit myself to just a few remarks here. 89 Kaminiates tells of Thessaloniki’s citizens who were sold to slave dealers in Tripoli, and were then resold again, destined for Ethiopia and further lands far to the south. 90 Thus their likelihood of ever coming back – contrary to what we know about the slaves al‐Wāthiq (r. 842–47) bought and then exchanged for Arab captives in 845 (see below) – was minimal. Indeed, some individuals had the chance of being bought by Christian subjects of the caliphate. Thus, St Elias the Younger, who was captured twice by Arabs was lucky enough to be sold to Christian masters. 91 Perhaps his life was spared and he shared the fate of future slaves because of his good looks, as his hagiographer indicates. Other Byzantine captives who had fallen into the hands of the enemy could access a better way of life thanks to their education. Such was the case with Cosmas the Hymnographer (*PmbZ* #4097 with all the versions of his *Lives*). Having been captured by the Arabs, he was taken to Damascus where he was bought by Sergios, the father of St John of Damascus. Cosmas became John’s teacher because Sergios wanted his son to study not only the books of the Saracens but also those of the Greeks. 92

### Serving a new master

Keeping in mind that sometimes the processes of ransom or exchange could take years, and not forgetting the harsh conditions of life in captivity, it comes as no surprise that sometimes Christian captives opted to convert to the religion of their new masters. That is what one of Emperor Leo III’s (r. 717–41) closest associates, Beser (*PmbZ* #1010), did. He is reported to have been taken captive by the Arabs, to have converted to Islam, and then to have come back to his homeland where he offered his services to the emperor. 93 During the siege of Amorion in 838, the Byzantine commander‐in‐chief Aetios sent two messengers to Constantinople beseeching rapid support in order to save the besieged city. However, they fell into Arab hands, leaving them limited options. Since the messengers were acquainted with the situation within the walls, they were considered even more valuable to the caliph. Letting them live was in this case obviously the better strategy for both sides. The Byzantine messengers converted to Islam, then al‐Muʽtaṣim gave them money, dressed them in new clothes, and paraded them along the walls of the besieged city with a view to lowering the morale of the enemy. 94

What opportunities defection could offer is shown by the story of Leo of Tripoli (*PmbZ* #24397). He was in probability not a captive but a renegade, yet choosing to convert and loyally serve his new Arab masters opened the way to his making a career as a naval commander, who repaid their trust by inflicting on his former countrymen one of the bitterest and most humiliating defeats they had ever suffered. 95 The same path was taken by a certain Luke, an Arab chieftain by the surname of Kaphiros (the Arab for non‐Muslim) and Apostates (the Greek for renegade or defector), who engaged in raids on southern Italy on various occasions around the year 1000 (*PmbZ* #24780). 96

We encounter a similar situation in the Balkans where the pagan Bulgars – though ready enough to maltreat their Christian captives – were at the same time prepared to use the military potential of Byzantine prisoners, as long as these were loyal and, one might suggest, did not try to promote Christianity at the expense of local paganism. 97 It was obviously no problem for Bulgaria’s Khan Krum (r. 803–14), who ordered the torture of Christian prisoners of war and the deportation of thousands of residents of Thrace, to trust a refugee – Constantine Patzikos (*PmbZ* #3920) – as one of his closest associates and to marry him to his sister. 98 Bulgar inscriptions dating from the reign of the same ruler relate to Byzantine *strategoi* (generals) by the names of Bardanes, Eanes (= Ioannes; John?), Kordyles, and Gregoras, who occupied high military offices in the Bulgar army and were second‐in‐command after members of the Bulgar elite. 99 What we still do not know for sure is whether these Byzantine generals were refugees, renegades, or prisoners of war who chose to offer their services to their new, heathen masters.

The readiness to use Byzantine captives for military service is attested even at the end of the tenth century. After the fall of Larissa in 986, the Tsar Samuel of Bulgaria (r. 977/97–1014) ordered its citizens to be resettled and then included in the recruits of his army. 100 However, there was always a probability that former imperial subjects would once again defect and rejoin their former countrymen. That is what Ašot (*PmbZ* #20650) did. A son of the governor of Thessaloniki Gregory Taronites (an Armenian by origin), Ašot was taken captive by the Bulgarians in the 990s, but his destiny as prisoner‐of‐war was more than favourable. Tsar Samuel married him to his daughter Miroslava and appointed him governor of Dyrrhachion, one of the most important fortresses in the Balkans. However, Ašot appears never to have been fully convinced by the Bulgarian victory and subsequently surrendered the city to Emperor Basil II (r. 976–1025). 101

### Exchanging captives

Kaminiates ends his eyewitness account by considering the two possible scenarios facing him as a captive, namely death or exchange. 102 Since the three occasions – Amorion in 838, Syracuse in 878, and Thessaloniki in 904 – on which this article focuses were major military losses that not only harmed the empire’s prestige but also resulted in taking thousands of imperial residents into captivity, negotiations initiated by the highest state authority needed to take place at the earliest opportunity. 103 Soon after the fall of Amorion, the emperor’s envoy Basil appeared before the caliph al‐Muʽtaṣim with presents and gifts, requesting negotiations for the return of the Byzantine prisoners. 104 As for the captives from Thessaloniki, a certain Symeon arrived in Thessaloniki and made Leo of Tripoli aware that the emperor was ready to exchange the Byzantine captives in return for the exact same number of Arab prisoners. At the time of these conversations, the Muslim troops were still within the sacked city. 105 Their commander, Leo of Tripoli, had already explained to Kaminiates what fate awaited him and his relatives:

We are bound for Syria, and when we get there I shall send you immediately to the city of Tarsos in Kilikia, to be held along with the others who are in detention there until the currently awaited exchange of prisoners takes place. You have as hostages for your safety those fellow Hagarenes of ours who have on numerous occasions been captured by the Romans. When they come home, you too will be set free and will regain, each one of you, his homeland. 106

For this purpose, either in 904 or in 905/906 one of the leading Byzantine diplomats of the time, Leo Choirosphaktes – his skill in negotiation already proven with the Bulgarians – departed for the east. 107 He was part of an embassy headed by the eunuch Basil, who himself remained in Tarsos, where he assembled prisoners from the borderland, while Leo was received by the caliph in Baghdad. 108 Unfortunately, Leo is extremely laconic in his correspondence and offers only a few words about his mission. In contrast to later envoys like Constantine Manasses, Theodore Metochites, or Nikephoros Gregoras, he does not share any details about the journey to Baghdad. Peter of Sicily was another diplomat from the Middle Byzantine period who is known to have travelled abroad with the task of freeing captives. He also wrote an account after his mission to the Paulicians in the late 870s, but he is more interested in narrating the history of the heretics than describing his stay in Tephrike and his journey to the eastern border, which he mentions just in passing. 109

In spite of all efforts, the diplomatic preparations for some exchanges – especially if they aimed to regulate the return of large numbers of prisoners, or the opposite side disagreed about some of the proposals made by Constantinople – could take years. 110 Byzantium accomplished the return of the captives taken in Syracuse in 884/5, some seven or eight years after their hometown’s sacking. 111 Approximately the same period was needed to determine the conditions under which the Amorian captives were to be set free. This was one of the large‐scale exchanges of prisoners between Byzantium and the Arabs. 112 It took place at the river Latmos near Seleucia in 845, and continued for four days. 113 Although two Byzantine governments – that of Theophilos and the subsequent one of his wife Theodora and their son Michael III (r. 842–67) – had worked for years towards liberating the captives, it seems that there were still disagreements between the two sides. The sack of Amorion may have been a huge blow to the empire’s prestige, yet the Byzantines had achieved their own victories against the Arabs, which brought thousands of Muslim captives into their hands. The events of 845 were therefore one of a series of exchanges in which the Byzantines held more prisoners than the Arabs had at their disposal. 114 Thus, the empire was not acting from a position of weakness. The main problem for the two powers was determining the kind of prisoners that were to be exchanged. Initially the Byzantines declined to accept an equal exchange and did not want to receive back old people and children. 115 According to Bar Hebraeus, the Arabs had their own objections – like the view of al‐Wāthiq’s father, who would not ‘admit that the Christians are of equal value with the Arabs when [weighed in] the balance for exchange’. 116 However, the new caliph abandoned the concerns of his predecessor and both sides finally agreed on an equal exchange soul for soul. The caliph even had to resort to buying slaves in order to match the quantity of prisoners possessed by the Byzantines. The number of Muslims who returned to the caliphate was estimated at 4,460 by al‐Ṭabarī, and thus we can postulate a similar number for the Byzantine captives released by the Arabs. 117

Two victories of the Ḥamdānid emir of Aleppo, Sayf al‐Dawla, over the Byzantines in 954 and 956 brought high‐ranking Byzantine officials into Arab captivity. Among them were Theodoulos Parsakoutenos (*PmbZ* #27993) – whose wife was sister of the general and future emperor Nikephoros II Phokas – one of his sons, and ibn al‐Balantis, probably a member of the Balantes family (*PmbZ* #22687). 118 The Byzantines only returned home a decade later, thus attracting the suspicion that it was the emir of Aleppo who had delayed an exchange or ransom. However, in around 963 Sayf al‐Dawla’s own cousin Abū Firās (*PmbZ* #20051) was taken captive by Theodore Parsakoutenos, whose father and brother were held by the Arabs as prisoners of war, and the emir had, therefore, to find a solution to settle the situation. 119 A series of Byzantine embassies headed to the east until the exchange finally took place on 23 June 966 on the banks of the upper Euphrates. 120 The emir of Aleppo received his cousin and various high officials back, and the members of the Phokas kin also returned home. Yet what happened seems to have been more than an exchange of elite prisoners of war. Yaḥyā of Antioch reports that the Arabs returned about 3,000 captives above the number held by the Byzantines, and that these were ransomed by Sayf al‐Dawla for 240,000 *nomismata*. 121

### The desire to return home

It is difficult to say whether the above‐mentioned prisoner of war Ašot chose to switch sides out of some kind of patriotism or whether his motives were more or less pragmatic. What we do know is that some captives were clearly susceptible to homesickness and so endeavoured to return one day to their native towns. 122 We may mention here two stories from the early medieval period. The first deals with the Christian captives whom the Avars resettled to the north of the Danube in the early seventh century. There they mixed with Avars and Bulgars, but their descendants preserved the faith of their ancestors and even wished to go back to the places whence their fathers originated. Thus, led by the Bulgar chieftain Kuber, they crossed the Danube and settled in the region of Thessaloniki. 123 Some two hundred years later, other Byzantine captives, who originated from Eastern Thrace but who had been resettled by the Bulgars to the north of the Danube, organized their escape to Byzantium. At their contacting Emperor Theophilos, he sent a fleet to the Lower Danube which brought them securely back to imperial territory. 124

## The social dimensions of captivity

### High lay officials

Once a city was sacked – especially if it was a major administrative and religious centre – the conquerors had to decide what the fate of the surviving citizens was going to be. Usually, they had a particular interest in capturing the noble and more prominent residents. 125 Of the destiny of Amorion’s inhabitants, we learn that on the caliph’s order those of higher social status were picked out. 126 Leo of Tripoli, the conqueror of Thessaloniki, followed a more or less similar strategy. On his behalf, prominent and wealthy citizens – among them high officials such as Niketas – were spared. 127 Later on, when the Arab fleet headed to Tarsos, the captured Byzantine *strategoi* sailed on Leo’s ship. It is hard to believe that he was motivated by respect for their dignity: providing for their security by keeping the most important prisoners in his ship, he could be sure that the lion’s share of potential ransom money would be preserved for himself. 128

In the ego‐accounts of the two Byzantines who were taken captive in the course of Arab campaigns against the empire – Theodosios the Monk and John Kaminiates – the figure of a Greek‐speaking captor occurs. 129 In Syracuse as well as in Thessaloniki, these soldiers appeared at places (the cathedral of Syracuse), or on occasions (the capture of the Kaminiates family by ‘Ethiopians’), where one might expect to capture objects or captives of high value. One may therefore suspect that the enemy commanders appointed soldiers with knowledge of the local language, whose task it was to secure both valuables and persons associated with high status – individuals, therefore, who were of more value alive as captives than dead. As for the shocked prisoners who survived, both Kaminiates and Theodosios speak graciously in their recollections about the captors who knew their language and treated them well, the first even calling the enemy soldier ‘a wonderful man’ and ‘saviour’. 130

At times, even high‐ranking prisoners of war – belonging, moreover, to the leading families of the empire – did not manage to return. In 954, the Ḥamdānid emir of Aleppo, Sayf al‐Dawla, defeated the *domestikos* Bardas Phokas the Elder near Germanikeia. The Arabs captured numerous patricians and close associates of Bardas, as well as his son Constantine (*PmbZ* #23841), and many soldiers were massacred. 131 Arab sources tell different stories about Constantine’s fate after the battle, but what is sure is that he died in captivity and never returned. Another prisoner of high social status who shared the same fate was the *domestikos* Melias (*PmbZ* #25042). He was captured during the siege of Amida in 973 and died in prison, most probably from his injuries or other health issues. 132

### High‐ranking church officials

One of the first questions the victorious Leo of Tripoli posed to John Kaminiates and his companions was whether his father was the local bishop. 133 By virtue of their authority, which had some congruence with that of high military commanders and governors, bishops enjoyed a particular power on a local and regional basis. 134 At times, when the enemy’s victory seemed sure they could become involved in negotiations, which may have spared them many of the atrocities faced by their captured countrymen. We know that in 838, when it became apparent that the city of Amorion was doomed, the bishop and three high officials led a delegation to the caliph and negotiated an evacuation of the city and its surrender. Their attempt to prevent what subsequently happened proved, however, to be in vain. 135 Furthermore, both Leo, bishop of Sicily (*PmbZ* #24414), and an anonymous governor of Calabria served as hostages after the Fāṭimid general Ǧaʽfar ibn ʽUbayd seized Oria (in Apulia) and signed a treaty with its residents. 136

An early tenth‐century letter by Arethas of Caesarea, himself an archbishop, contains an interesting account of the different kinds of behaviour that high clergymen displayed in the face of danger. The author – not unexpectedly – did not hesitate to pronounce his moral verdict: he blamed Stephen, bishop of Adrianople (*PmbZ* #27250), who fled from the Bulgarians hidden in a basket, thus disregarding his previous oaths. Not that this saved him, for he was captured and suffered maltreatment at the hands of his captors. 137 Arethas tells the story of another bishop from Thrace, Euthymios (*PmbZ* #21931), whose diocese had its seat in Rhousion. He was also captured, though – unlike Stephen – Euthymios did *not* obey the Bulgarians’ orders but rather withstood their threats. By doing so, he was honoured by both Bulgarians and Byzantines. However, it is not clear under what circumstances he died. 138

Usually after a major urban centre had been sacked, the local bishop followed his flock into captivity. Thus the bishop of Rhegion (Reggio di Calabria) was taken captive by the Arabs in 901, together with 17,000 people. 139 According to al‐Ṭabarī, in 806 the general in ʽAbbāsid service, Ḥumayd ibn Maʽyūf al‐Ḥajūrī, launched a raiding campaign against Cyprus. Sixteen thousand of its inhabitants are said to have been enslaved and brought to Caliph Hārūn al‐Rashīd. At his orders they were sold, and ‘the bishop of Cyprus fetched two thousand dīnārs’. 140 What the Arab historian does not say precisely is whether the high clergyman was ransomed or sold into slavery. In the encounters between Christianity on the one hand, and Islam and paganism on the other, high church officials wielded a strongly symbolic power for their coreligionists and captors alike, and were regarded as the very embodiment of Christian worship. Theodosios the Monk thus relates in his witness account that he and the bishop of Syracuse took off their vestments and hid in the cathedral, obviously wishing to deceive the Muslim soldiers. 141 They were safe once the Arabs – one of whom spoke Greek – had seized the sacred objects. 142 What was to follow were long months of captivity, first in the captured Syracuse, and then in Palermo. In Palermo prison they met another bishop, of Malta, who had been living in captivity for eight years. 143 It escapes our knowledge whether the two bishops and Theodosios were among the prisoners who were set free in 884/5.

Some bishops became victims of the enemy’s religious zeal. In 666/7, at the high point of the Arab conquest in the east, two high church officials lost their lives. Theophanes says that, during the campaign of Busir in Syria, two bishops – of Apameia and Emesa – were put to death. The latter was burned alive. 144 The attitude of the pagan Bulgars to high Christian clergymen who fell into their hands was also far from benevolent. After the sack of Debeltos, the Bulgars organized a resettlement of the local Greek population, which also involved the bishop. 145 According to the *Synaxarion of the Constantinopolitan Church*, his name was Peter, and he was later killed in captivity. 146 A sermon commemorating the names of those Christians who were murdered while in Bulgarian captivity mentions two other Byzantine bishops who shared the fate of Peter of Debeltos: these were Manuel of Adrianople and Leo of Nicaea. 147

### Women

Once an enemy’s resistance had been overwhelmed, victors turned their attention to looting the property of the defeated and to a certain group of civilians that were usually of great interest to them – the women. 148 Among these, young women, and especially virgins, were considered a highly prized trophy. Thus, after the sacking of Amorion, the raiding soldiers of the caliph focused on nunneries, where they knew what kind of prey was to be expected. According to Syrian chroniclers, thousands of virgins fell into the hands of the caliph’s Turkic and Moorish soldiers, not even counting those who were killed. 149 As for married women, al‐Muʽtaṣim gave the order that they be sold in groups of five or ten, but not to separate children from their parents. 150 A similar interest is attested for Leo of Tripoli’s fighters, the majority of whose captives were exclusively young men and women, and children. 151 The fate of captured women was often slavery or forced marriage. If they were of enormous beauty, they were considered especially valuable, as we learn from the story of a young woman from the southern Peloponnese. Because she was very beautiful, her captors are said to have kept her as a prize for their emir. 152

Some of the women captured in Thessaloniki in 904 were bought by men in Bolbos, who had already made an early appearance in the sacked city. 153 With regard to their particular interest in women, it is more likely that they were Byzantine slave traders trying to make some profit from the misfortune of their countrywomen than local inhabitants seeking to pay the ransoms of their female relatives. The daughters of Christian high officials taken into captivity seem to have been regarded as an especially valuable asset, as evidenced by the fact that the daughter of the patrician Gregory was preserved as a trophy for the Arab chieftain (*PmbZ* #2345a).

In terms of numbers, we can assume that women comprised at least half of civilian captives, if not more. 154 It is, however, difficult to reconstruct the captivity experience of individual women since almost all are anonymous. Even the name of the above‐mentioned patrician’s daughter or that of Kaminiates’ sister‐in‐law, who was sold to slave traders on Crete, remain unknown. 155 Female captives can lose their anonymity in hagiographical works – consider, for example, St Theoktiste of Lesbos (*PmbZ* #8027) 156 – but the question about reality and fiction in saints’ *Lives* remains a relevant one. 157 We know the name of a certain Maria, wife of Peter, both of whom suffered a martyr’s death in Bulgarian captivity, and who were included in a sermon honouring those Christians who died in Bulgaria. 158 In Maria’s case, however, the text does not give any details on her life as a captive. Another female captive whom we know by name was the mother of Basil I. Here one needs to make two clarifications. First, although her story is preserved in the *Vita Basilii* (the biography of her son), she still remains anonymous in the account of the Byzantine historian. 159 We only know of her name (Pankalo) thanks to the *Book of Ceremonies* listing the burial places of all the members of the Macedonian dynasty to date. 160 Second, the reason for her story being narrated in a historiographical work was not an interest in her individual experience of captivity but the simple fact that her son, who lived as a captive during his childhood, was later to become emperor. 161

## Conclusions

The price for the survival of the ‘empire that would not die’ during the transition from Late Antiquity to the early Middle Ages was paid to a certain degree by those of its subjects who were spared by their enemies but had to spend their futures in captivity. What is striking in the period under consideration is the sheer volume of such forced mobility, the duration of the state of captivity for many prisoners (sometimes up to ten years are attested), and the distances that separated these people from their homes. Thousands of Byzantines suffered enforced mobility in this context.

The majority of these people will remain anonymous, especially if one tries to study what happened further down the social ladder and if one endeavours to look beyond the anonymous mass of the *aichmalotoi* of medieval Greek texts. However, even the fate of high‐status captives and their families poses more questions than their own recollections can answer. Medieval historiography usually sheds little light on individuals and keeps its focus on events such as the sack of cities, the outcome of battles, and the exchange of prisoners of war. More helpful in this regard proves to be hagiography, with its holy protagonists who not only helped captives but sometimes experienced captivity themselves. For the time under consideration there are only two Byzantine ego‐accounts (by John Kaminiates and Theodosios the Monk) that offer us the opportunity to follow the individuated fates of captives in greater detail.

It is clear that ransom, enslavement, serving a new master, and undergoing exchange were among the usual fates of Byzantine captives. Some of these scenarios could facilitate a better life far from home; others made redemption possible. Without a doubt, at least theoretically speaking, a higher social status offered better chances of surviving the hazards of forced mobility and, finally, the possibility of a way back home. However, war has always had its own rules and logic, thus making any generalization or attempt to place life in captivity into a theoretical framework or schema a tricky task. In cases of forced mobility, the last word went to the victors, who ultimately had the agency and authority to decide the fates of their captives.

## Footnotes

^1^
Isauria: Theophanes, *Chronographia* A.M. 6142, ed. C. de Boor, *Theophanis chronographia* (Leipzig, 1883), p. 344, ll. 19–21. Reggio di Calabria: John the Deacon, *Translation of St Severinus*, c. 1, ed. G. Waitz, in *MGH SRL* 1 (Hanover, 1878), p. 453, ll. 6–10. Thessaloniki: John Kaminiates, *On the Capture of Thessaloniki*, c. 73.2, ed. G. Böhlig, *Ioannis Caminiatae de expugnatione Thessalonicae*, Corpus Fontium Historiae Byzantinae 4 (Berlin and New York, 1973), p. 62. Tiriolo: *Chronicle of Cambridge arab*., *a*. 6438, trans. M. Amari, *Biblioteca arabo‐sicula. Versione italiana*, 2 vols (Turin and Rome, 1880; repr. Milan, 1982), vol. 1, p. 284. For other sources reporting on 18,000 captives, see *PmbZ* # 26951 (full *PmbZ* citation in note 4 below).

^2^
P. Cosme *et al*. (eds), *Le récit de guerre comme source d’histoire, de l’Antiquité à nos jours* (Besançon, 2022).

^3^
For the various forms of forced migration, see the typology offered by Y. Stouraitis, ‘Migrating in the Medieval East Roman World, ca. 600–1204’, in J. Preiser‐Kapeller *et al*. (eds), *Migration Histories of the Medieval Afroeurasian Transition Zone. Aspects of Mobility between Africa, Asia and Europe, 300*–*1500 c.e* . (Leiden and Boston, 2020), pp. 141–65. On forced migration in Byzantium, see also C. Rapp, ‘Zwangsmigration in Byzanz: Kurzer Überblick mit einer Fallstudie aus dem 11. Jahrhundert’, in Th. Ertl (ed.), *Erzwungene Exile. Umsiedlung und Vertreibung in der Vormoderne (500 bis 1850)* (Frankfurt and New York, 2017), pp. 59–79, as well as the case studies discussed in C. Rapp and J. Preiser‐Kapeller (eds), *Mobility and Migration in Byzantium: A Sourcebook* (forthcoming).

^4^
References to the *Prosopography of the Middle Byzantine Period* (hence *PmbZ*) are based on R.‐J. Lilie *et al*. (eds), *Prosopographie der mittelbyzantinischen Zeit*, 14 vols (Berlin and New York, 1998–2013), also available online at https://www.degruyter.com/database/pmbz/html?lang=de.

^5^
In the field of Byzantine studies, there has been valuable research on captivity (see notes 8, 9 and 103 below). However, these studies often combine the fate of Byzantine captives abroad with the destiny of foreign captives in Byzantium. Although one can appreciate the value of such a comparative approach, separating outgoing from incoming migration can clarify analysis of the ways in which Byzantines experienced captivity and forced migration. Another peculiarity of overviews offered by Byzantinists is that they tend to neglect the account of Theodosios the Monk, one of the central texts on this topic. The present paper aims to rectify that and will therefore use Theodosios’ valuable recollections as one of the seminal sources on forced migration as experienced by Byzantines.

^6^
A. Classen (ed.), *Incarceration and Slavery in the Middle Ages and the Early Modern Age. A Cultural‐Historical Investigation of the Dark Side in the Pre‐Modern World* (Lanham, 2021); M.F. Lopes de Barros and C. Almagro Vidal (eds), *Forms of Unfreedom in the Medieval Mediterranean* (Évora, 2021). Another major issue was religion. Since this necessitates a more comprehensive study, I plan to deal with the religious aspects of the experience of captivity elsewhere.

^7^
M. Lykaki, ‘L’économie du pillage et les prisonniers de guerre: Byzance, VIIe–Xe siècle’, in R. Keller and L. Sarti (eds), *Pillages, tributs, captifs. Prédation et sociétés de l’Antiquité tardive au haut Moyen Âge* (Paris, 2018), pp. 89–102; J. Haldon, *Warfare, State and Society in the Byzantine World, 565*–*1204* (London, 1999), pp. 244–6.

^8^
On Byzantines held captive by enemies as well as foreign captives in Byzantium, see S. Patoura, *Οἱ αἰχμάλωτοι ὡς παράγοντες ἐπικοινωνίας καὶ πληροφόρησης (4ος*–*10ος αἰ.)* (Athens, 1994); M. Lykake, ‘Οι αιχμάλωτοι πολέμου στη Βυζαντινή Αυτοκρατορία (6ος–11ος αι.): Εκκλησία, Κράτος, διπλωματία και κοινωνική διάσταση’, Ph.D. thesis, Εθνικόν και Καποδιστριακόν Πανεπιστήμιον Αθηνών, École Pratique des Hautes Études IVe Section, Paris (2016); A. Kolia‐Dermitzaki, ‘Some Remarks on the Fate of Prisoners of War in Byzantium (9th–10th Centuries)’, in G. Cipollone (ed.), *La liberazione dei ‘captivi’ tra Cristianità e Islam. Oltre la crociata e il ğihād: tolleranza e servizio umanitario* (Vatican City, 2000), pp. 583–620; Y. Hristov, ‘Prisoners of War in Early Medieval Bulgaria (Preliminary Remarks)’, *Studia Ceranea* 5 (2015), pp. 73–105.

^9^
On terminology, see Y. Rotman, *Byzantine Slavery and the Mediterranean World*, trans. J.M. Todd (Cambridge, MA and London, 2009), pp. 27–8; J.W. Brodman, ‘Captives or Prisoners: Society and Obligation in Medieval Iberia’, *Anuario de Historia de la Iglesia* 20 (2011), pp. 201–19; N. Lenski, ‘Slavery in the Byzantine Empire’, in C. Perry *et al*. (eds), *The Cambridge World History of Slavery*, vol. 2: *ad 500–AD 1420* (Cambridge, 2021), pp. 453–81, esp. pp. 457–9; A. Niewiński, ‘The Treatment of Prisoners of War in the Middle Ages: Western European Examples’, *Roczniki Humanistyczne* 67.2 (2019), pp. 7–41, esp. p. 7.

^10^
Brodman, ‘Captives or Prisoners’, pp. 202–3 and 207–8; M. Bennett and K. Weikert, ‘The State of Play: Medieval Hostageship and Modern Scholarship’ in *iidem* (eds), *Medieval Hostageship, c.700–c.1500. Hostage, Captive, Prisoner of War, Guarantee, Peacemaker* (New York and Abingdon, 2017), pp. 1–14.

^11^
Rotman, *Byzantine Slavery*, p. 28. As well as the term *aichmalotos*, there are designations such as *andrapodon*, *desmios* and *desmotes*, cf. D. Letsios, ‘Die Kriegsgefangenschaft nach Auffassung der Byzantiner’, *Byzantinoslavica* 53 (1992), pp. 213–27, esp. p. 215; Ch. Papavarnavas, ‘Imprisoned Martyrs on the Move: Reading Holiness in Byzantine Martyrdom Accounts’, *Byzantinische Zeitschrift* 114.3 (2021), pp. 1241–62, esp. pp. 1243–4.

^12^
For a general overview, see D. Sullivan, ‘Byzantine Fronts and Strategies 300–1204’, in Y. Stouraitis (ed.), *A Companion to the Byzantine Culture of War, ca. 300*–*1204* (Leiden and Boston, 2018), pp. 259–307. I use the term ‘Bulgar’ to designate the earlier pagan and non‐Slavic‐speaking people, and ‘Bulgarian’ for the inhabitants of the land who became Christianized in the 860s, and whose official and liturgical language was Slavonic.

^13^
J. Haldon, *The Empire that Would not Die. The Paradox of Eastern Roman Survival, 640*–*740* (Cambridge, MA and London, 2016); W. Treadgold, *The Byzantine Revival 780*–*842* (Stanford, 1988).

^14^
V. Christides, *The Conquest of Crete by the Arabs (ca. 824). A Turning Point in the Struggle between Byzantium and Islam* (Athens, 1984); M. Leontsini, ‘The Byzantine and Arab Navies in the South Aegean and Crete: Shipping, Mobility and Transport (7th–9th c.)’, *Graeco‐Arabica* 12 (2017), pp. 171–234.

^15^
Y. Rotman, ‘Migration and Enslavement: A Medieval Model’, in Preiser‐Kapeller *et al*. (eds), *Migration Histories*, pp. 387–412.

^16^
St. Efthymiadis, ‘Chrétiens et sarrasins en Italie méridionale et en Asie Mineure (IXe–XIe siècle). Essai d’étude comparée’, in A. Jacob *et al*. (eds), *Histoire et culture dans l’Italie byzantine. Acquis et nouvelles recherches* (Rome, 2006), pp. 589–618, esp. pp. 593–5.

^17^
Ch. Messis, ‘La mémoire du “je” soufrant: construire et écrire la mémoire personnelle dans les récits de captivité’, in P. Odorico *et al*. (eds), *L’écriture de la mémoire. La littérarité de l’historiographie. Actes du IIIe colloque international philologique, Nicosie, 6*–*7*–*8 mai 2004 organisé par l’E.H.E.S.S. et l’Université de Chypre* (Paris, 2006), pp. 107–46; A.J. Goldwyn, *Witness Literature in Byzantium. Narrating Slaves, Prisoners, and Refugees* (Cham, 2021). Goldwyn’s major contribution is, in my view, his wish to ‘humanize’ the story of Byzantine captives and to redirect scholarly attention towards the emotional dimension of the Byzantine experience of captivity. However, a brief remark on the use of ‘Holocaust literature’ as a method for analysing Byzantine witness accounts seems relevant here. Although one cannot deny the extent of the impact that wars and raids caused across the borders in the east and the west, conflicts between Byzantium and its neighbours were marked by violence based on *religion*. Therefore, at least in theoretical terms, captives had the chance of survival by opting to change their religion. This contrasts with the approach by the Nazis, racists, or white supremacists towards Jews or people of colour, based on ethnic or racial origin.

^18^
Of the original Greek text of Theodosios’ letter only the beginning has survived. The preserved part is published by C.O. Zuretti, ‘Ἰταλοελληνικά’, in *Centenario della nascita di Michele Amari*, 2 vols (Palermo, 1910; repr. Palermo, 1990, ed. R. Giuffrida), vol. 1, pp. 165–84. In the late sixteenth or the early seventeenth century, however, the entire Greek original was translated into Latin, first by Josaphat Atzale/Azzale and then by Agostino Fiorito, thus allowing us to reconstruct Theodosios’ recollections of his experience as a captive. Both translations are published by G. Satta, *La conquista araba di Siracusa con l’Epistola di Teodosio monaco in appendice* (Augusta, 2007), pp. 49–69. When referring to the two Latin translations I will use the designation (Az.) for Atzale and (Fi.) for Fiorito. An Italian translation can be found in L. Santagati, *Storia dei Bizantini di Sicilia* (Caltanissetta, 2012), pp. 274–82. On the history of this interesting text and its transmission, see, apart from Satta’s study, also C. Rognoni, ‘Au pied de la lettre? Réflexions à propos du témoignage de Théodose, moine et *grammatikos*, sur la prise de Syracuse en 878’, in A. Nef and V. Prigent (eds), *La Sicile de Byzance à Islam* (Paris, 2010), pp. 205–28; *eadem*, ‘Una rappresentazione letteraria del confronto tra Bizantini e Arabo‐Musulmani nella Sicilia del IX secolo. Note in margine all’epistola di Teodosio monaco’, in S. Modeo *et al*. (eds), *La Sicilia del IX secolo tra Bizantini e musulmani. Atti del IX convegno di studi* (Caltanisetta, 2013), pp. 195–208; V. von Falkenhausen, ‘La conquista di Siracusa (878) nella memoria storica di Costantinopoli’, in M. Palma and C. Vismara (eds), *Per Gabriella. Studi in ricordo di Gabriella Braga*, 2 vols (Cassino, 2013), vol. 2, pp. 835–48.

^19^
Engl. translation in D. Frendo and A. Fotiou, *John Kaminiates, The Capture of Thessaloniki. Translation, Introduction and Notes* (Perth, 2000). Although A.P. Kazhdan, ‘Some Questions Addressed to the Scholars Who Believe in the Authenticity of Kaminiates’ “Capture of Thessalonica”’, *Byzantinische Zeitschrift* 71 (1978), pp. 301–14, called into question the authenticity of the account, research on this topic over the last four decades appears to prove the historicity of Kaminiates’ work. On the account, its author, and the arguments against Kazhdan’s concerns, see P. Odorico, ‘Les trois visages de la même violence: les trois prises de Thessalonique’, in Odorico *et al*., *L’écriture de la mémoire*, pp. 147–79; *idem*, *Jean Caminiatès, Eustathe de Thessalonique, Jean Anagnostès. Thessalonique: Chroniques d’une ville prise. Textes présentés et traduits du grec* (Toulouse, 2005), pp. 7–53; L. Neville, ‘Pity and Lamentation in the Authorial Personae of John Kaminiates and Anna Komnene’, in St. Constantinou and M. Meyer (eds), *Emotions and Gender in Byzantine Culture* (Cham, 2019), pp. 65–92.

^20^
Odorico, ‘Visages’, pp. 154–6; *idem*, *Caminiatès*, pp. 13–22; Satta, *Conquista*, pp. 10–11; G. Strano, ‘Storia e modelli letterari nella Presa di Tessalonica di Giovanni Caminiata’, in A. Rigo *et al*. (eds), *Vie per Bisanzio. VII Congresso nazionale dell’Associazione Italiana di Studi Bizantini. Venezia, 25*–*28 novembre 2009*, vol. 1 (Bari, 2013), pp. 61–74, esp. pp. 64–5 and 71–4. *Contra* Rognoni, ‘Réflexions’ pp. 211–12 and 217, n. 48, who argues that Theodosios was one of the captives redeemed in 884/5 and that he wrote his letter following his release. She considers the text to be an attempt by the author to downplay the failure of Emperor Basil I (r. 867–86) to save Syracuse, by shifting the focus onto the savageness of the Arabs and the harshness of captivity.

^21^
M. Reitmayer, *Entführung und Gefangenschaft. Erfahrene Unfreiheit in gewaltsamen Konflikten im Spiegel spätmittelalterlicher Selbstzeugnisse* (Konstanz, 2021).

^22^
Goldwyn, *Witness Literature*, pp. 162–65 and 195.

^23^
R. Khouri, ‘Ανεπίσημες ανταλλαγές, εξαγορές και απελευθερώσεις Βυζαντινών και Αράβων αιχμαλώτων’, *Graeco‐Arabica* 4 (1991), pp. 109–13, esp. p. 109; Haldon, *Warfare, State and Society*, pp. 245–7.

^24^
The text was edited by V. Vasil’evskij and P. Nikitin, *Skazanija o 42 amorijskich mučenikach i cerkovnaja služba im*, Zapiski Imperatorskoj akademii nauk 8, Po istoriko‐filologičeskomu otděleniju 7.2 (St Petersburg, 1905). See also D. Krausmüller, ‘Establishing the Relationship between the Passiones Δ, Β and Γ of the Forty‐Two Martyrs of Amorium’, *Rivista di Studi Bizantini e Neoellenici*, ns 56 (2019), pp. 41–56; S. Kotzampase, ‘Το μαρτύριο των μβ´ μαρτύρων του Αμορίου. Αγιολογικά και υμνολογικά κείμενα’, *Επιστημονική Επετηρίδα της Φιλοσοφικής Σχολής, Αριστοτελείο Πανεπιστήμιο Θεσσαλονίκης, Περίοδος Β´, Τεύχος Τμήματος Φιλολογίας* 2 (1992), pp. 109–53. For a historical analysis of its data, see A. Kolia‐Dermitzaki, ‘The Execution of the Forty‐two Martyrs of Amorion: Proposing an Interpretation’, *Al‐Masāq* 14.2 (2002), pp. 141–62.

^25^
A. Lampadaridi *et al*. (eds), *Byzantine and Medieval History as Represented in Hagiography* (Uppsala, 2022).

^26^
Y. Stouraitis, ‘Historicity, Agency, and Ideology: The Story of the Sack of Amorion between Reality and Fiction’ (forthcoming).

^27^
Rotman, *Byzantine Slavery*, pp. 44 and 50–1.

^28^
Brodman, ‘Captives or Prisoners’, p. 212.

^29^
For the frontier in the east, see A. Asa Eger, *The Islamic‐Byzantine Frontier. Interaction and Exchange among Muslim and Christian Communities* (London and New York, 2015), esp. pp. 246–63.

^30^
Cf. *Life of St Mary the Younger*, c. 11, ed. H. Delehaye and P. Peeters, in *Acta Sanctorum*, *Novembris* IV (Brussels, 1925), p. 697 B.

^31^
Efthymiadis, ‘Chrétiens et sarrasins’, pp. 606–8 and 613–14.

^32^
George the Monk, *Chronicle* IX.43, ed. C. de Boor, *Georgii Monachi chronicon*, 2 vols (Leipzig, 1904), vol. 2, pp. 797–8; Michael the Syrian, *Chronicle* XII.20, trans. J.‐B. Chabot, *Chronique de Michel le Syrien, Patriarche Jacobite d’Antioche (1166*–*1199)*, 4 vols (Paris, 1905), vol. 3, p. 99.

^33^
Theodosios the Monk, ed. Zuretti, p. 167.

^34^
Kaminiates, c. 58.7, ed. Böhlig, p. 50.

^35^
Theodosios the Monk, ed. Satta, pp. 56, ll. 13–21 (Az.) and 66, ll. 24–34 (Fi.). On prisons, see Ch. Papavarnavas, *Gefängnis als Schwellenraum in der byzantinischen Hagiographie. Eine Untersuchung früh‐ und mittelbyzantinischer Märtyrerakten* (Berlin and Boston, 2021).

^36^
Kaminiates, cc. 67–9, ed. Böhlig, pp. 56–9.

^37^
Theodosios the Monk, ed. Satta, pp. 57, ll. 22–36 (Az.) and 68, ll. 1–10 (Fi.).

^38^
Theophanes Continuatus III.35, ed. M. Featherstone and J. Signes Codoñer, *Chronographiae quae Theophanis Continuati nomine fertur libri I*–*IV*, Corpus Fontium Historiae Byzantinae 53 (Berlin and Boston, 2015), pp. 188–90, ll. 1–9.

^39^
Patoura, *Αἰχμάλωτοι*, p. 26; Niewiński, ‘Treatment’, p. 18.

^40^
Michael the Syrian XII.20, trans. Chabot, vol. 3, p. 100; Bar Hebraeus, *Chronography*, in *idem, The Chronography of Gregory Abû’l‐Faraj 1225*–*1286, the Son of Aaron, the Hebrew Physician Commonly Known as Bar Hebraeus, Being the First Part of His Political History of the World*, trans. E. Wallis Budge (London, 1932; repr. Amsterdam, 1976), p. 137.

^41^
Al‐Ṭabarī, *History*, in *idem, The History of al‐Ṭabarī (Ta’rīkh al‐rusul wa’l‐mulūk)*, ed. E. Yar‐Shater, 40 vols (Albany, 1989–2007), vol. 33, trans. C.E. Bosworth, pp. 118–19.

^42^
On Emir Sayf al‐Dawla, see M. Canard, ‘Histoire de la Dynastie des H’amdanides de Jazîra et de Syrie’, Ph.D. thesis, Université d’Alger (1949), pp. 579–663.

^43^
John Skylitzes, *Synopsis*, ed. H. Thurn, *Ioannis Scylitzae synopsis historiarum*, Corpus Fontium Historiae Byzantinae 5 (Berlin and New York, 1973), pp. 241–2, ll. 30–46.

^44^
Al‐Ṭabarī, *History*, vol. 24, trans. D.S. Powers, p. 63. For the date, see *PmbZ* # 10840.

^45^
Al‐Ṭabarī, *History*, vol. 33, trans. Bosworth, p. 116.

^46^
Theodosios the Monk, ed. Satta, p. 54, l. 9–p. 55, l. 12 (Az.) and p. 64, l. 8–p. 65, l. 15 (Fi.).

^47^
Theodosios the Monk, ed. Satta, pp. 53, ll. 21–6 and 54, ll. 1–9 (Az.) and 63, ll. 7–10 and p. 63, l. 32–p. 64, l. 7 (Fi.).

^48^
Kaminiates, c. 59, ed. Böhlig, pp. 51–2; Theophanes Continuatus VI.i.21, ed. I. Bekker, *Theophanes Continuatus, Ioannes Cameniata, Symeon Magister, Georgius Monachus* (Bonn, 1838), p. 368, ll. 6–16.

^49^

*Life of St Cosmas the Hymnographer*, ed. Th. Detorakes, ‘Ἀνέκδοτος Βίος Κοσμᾶ τοῦ Μαϊουμᾶ’, *Ἐπετηρὶς Ἑταιρείας Βυζαντινῶν Σπουδῶν* 41 (1974), pp. 259–96, at pp. 272–3, ll. 274–80.

^50^

*Martyrion of St Nicholas of Bounaine*, ed. D. Sophianos, *Ἅγιος Νικόλαος ὁ ἐν Βουναίνῃ. Ἀνέκδοτα ἁγιολογικὰ κείμενα. Ἱστορικαὶ εἰδήσεις περὶ τῆς μεσαιωνικῆς Θεσσαλίας (Ι´ αἰών)* (Athens, 1972), pp. 141–3, ll. 92–147.

^51^
P. Buresi, ‘Captifs et rachat de captifs. Du miracle à l’institution’, *Cahiers de civilisation médiévale* 50 (2007), pp. 113–30, esp. pp. 115–16; Bennett and Weikert, ‘Medieval Hostageship’, pp. 4–5; Kolia‐Dermitzaki, ‘Amorion’, p. 150.

^52^
Theophanes Continuatus VI.i.9, ed. Bekker, p. 358, ll. 4–7.

^53^
A. Vasiliev, *Byzance et les Arabes*, 2 vols (Brussels, 1950–1968), vol. 2.2, p. 61.

^54^
Kaminiates, c. 77.5–7, ed. Böhlig, p. 66.

^55^
Joseph Genesios, *On the Reigns of the Emperors* III.11, ed. A. Lesmüller‐Werner and H. Thurn, *Iosephi Genesii regum libri quattuor*, Corpus Fontium Historiae Byzantinae 14 (Berlin and New York, 1978), pp. 44–5, ll. 42–60; *Martyrion of the 42 Martyrs of Amorion* (BHG 1210), ed. A. Vasil’ev, *Grečeskij tekst žitija soroka dvuch amorijskich mučenikov: po rukopisi Parižskoj Nacional’noj Biblioteki No 1534*, Zapiski Imperatorskoj akademii nauk 8, Po istoriko‐filologičeskomu otděleniju 3.3 (St Petersburg, 1898), p. 10, ll. 13–25; Bar Hebraeus, trans. Wallis Budge, pp. 135–6.

^56^
Theophanes, ed. de Boor, p. 490, ll. 21–7; I. Dujčev, ‘La chronique byzantine de l’an 811’, *Travaux et Mémoires* 1 (1965), pp. 205–54, at pp. 210–12, ll. 15–26.

^57^

*Scriptor Incertus*, ed. F. Iadevaia, *Scriptor Incertus. Testo critico, versione italiana, note e indici*, 2nd edn (Messina, 1997), pp. 43–5, ll. 136–53 and 168–90.

^58^
Theodosios the Monk, ed. Satta, pp. 55, ll. 12–19 (Az.) and 65, ll. 16–25 (Fi.).

^59^
Kaminiates, cc. 46.13 and 47–8, ed. Böhlig, pp. 41–3.

^60^
Kaminiates, cc. 55.11–13 and 56.1, ed. Böhlig, p. 58.

^61^
Kaminiates, c. 58, ed. Böhlig, pp. 50–1.

^62^
Al‐Ṭabarī, *History*, vol. 33, trans. Bosworth, pp. 114–15; Bar Hebraeus, trans. Wallis Budge, p. 137.

^63^
For a commentary, see Lensky, ‘Slavery’, pp. 461–2.

^64^
Y. Rotman, ‘Captives and Redeeming Captives: The Law and the Community’, in B. Isaac and Y. Shahar (eds), *Judaea‐Palaestina, Babylon and Rome: Jews in Antiquity* (Tübingen, 2012), pp. 227–48, esp. pp. 234–7; C. Rapp, *Holy Bishops in Late Antiquity. The Nature of Christian Leadership in an Age of Transition* (Berkeley *et al*., 2005), pp. 228–32; Brodman, ‘Captives or Prisoners’, p. 206.

^65^
Al‐Ṭabarī, *History*, vol. 38, trans. F. Rosenthal, p. 97.

^66^

*Life of St Neilos of Rossano*, c. 70, ed. G. Giovanelli, *Βίος καὶ πολιτεία τοῦ ὁσίου πατρὸς ἡμῶν Νείλου τοῦ Νέου. Testo originale greco e Studio introduttivo* (Grottaferrata, 1972), p. 110.

^67^

*Life of St Neilos of Rossano*, cc. 68–9, ed. Giovanelli, pp. 108–9.

^68^
The monastery probably lay in the border zone between Byzantium and Georgia, cf. *PmbZ* #24838.

^69^
H. Zimmermann, *Papsturkunden 896*–*1046*, vol. 1: *896*–*996* (Vienna, 1984), pp. 23–4, no. 12; J. Mabillon, *Vetera Analecta* (Paris, 1723), p. 428.

^70^
Nicholas Mystikos, *Letter* 145, ed. R.J.H. Jenkins and L.G. Westerink, *Nicholas I Patriarch of Constantinople, Letters. Greek Text and English Translation*, Corpus Fontium Historiae Byzantinae 6 (Washington, DC, 1973), pp. 458–60.

^71^
Kaminiates, c. 62.5, ed. Böhlig, pp. 53–4.

^72^
H. Grieser and N. Priesching (eds), *Gefangenenloskauf im Mittelmeerraum: ein interreligiöser Vergleich. Akten der Tagung vom 19. bis 21. September 2014 an der Universität Paderborn* (Hildesheim, 2015); Buresi, ‘Captifs et rachat de captifs’, pp. 113–30.

^73^
For Michael Aichmalotos, see J. Lefort *et al*., *Actes d’Iviron*, vol. 1: *Des origines au milieu du XIe siècle. Édition diplomatique*. *Texte* (Paris, 1985), pp. 198, l. 18 and 199, ll. 16–17, no. 17; for Leon Aichmalotos, see F. Trinchera, *Syllabus graecarum membranarum* (Naples, 1865), p. 6, no. 8; for Pothetos Aichmalotos or Aichmalotou, see A.K. Orlandos and L. Branouses, *Τὰ χαράγματα τοῦ Παρθενῶνος ἤτοι ἐπιγραφαὶ χαραχθεῖσαι ἐπὶ κιόνων τοῦ Παρθενῶνος κατὰ τοὺς παλαιοχριστιανικοὺς καὶ βυζαντινοὺς χρόνους* (Athens, 1973), p. 10, no. 19.

^74^

*Actes d’Iviron*, no. 16, ed. Lefort *et al*., vol. 1, p. 192, ll. 24–32.

^75^
Cf. J.‐C. Cheynet, *Pouvoir et contestations à Byzance (963*–*1210)* (Paris, 1990).

^76^
The Paulicians were followers of a religious and at the same time extremely bellicose sect persecuted by the Byzantine government in the early half of the ninth century. Due to the imperial policy, they defected to the Arabs and became a real threat to Constantinople, cf. C. Dixon, *The Paulicians. Heresy, Persecution and Warfare on the Byzantine Frontier, c. 750*–*880* (Leiden and Boston, 2022).

^77^
Theophanes Continuatus IV.23, ed. Featherstone and Signes Codoñer, p. 252, ll. 17–26. For the question about the historicity of this episode, see *PmbZ* #42.

^78^
Stephen of Tarōn, *Universal History* III.37, trans. T. Greenwood, *The Universal History of Stepʿanos Tarōnecʿi. Introduction, Translation, and Commentary* (Oxford, 2017), p. 300; Yaḥyā of Antioch, *History*, ed. I. Kratchkovsky and A. Vasiliev, *Histoire de Yahya‐ibn‐Saʿïd d’Antioche, continuateur de Saʿïd‐ibn‐Bitriq*, 2 fasc. Patrologia Orientalis 18.5 and 23.3 (Paris, 1924–32), fasc. 2, pp. 455–6.

^79^
Theophanes, ed. de Boor, p. 456, ll. 15–22.

^80^
Theophanes Continuatus III.34, ed. Featherstone and Signes Codoñer, p. 188, ll. 25–9; Bar‐Hebraeus, trans. Wallis Budge, p. 138.

^81^
Genesios III.12, ed. Lesmüller‐Werner and Thurn, p. 46, ll. 87–95.

^82^
Theophanes Continuatus IV.22, ed. Featherstone and Signes Codoñer, p. 248, ll. 18–27.

^83^
Leo the Deacon, *History* V.1, ed. K.B. Hase, *Leonis Diaconi Caloënsis historiae libri decem* (Bonn, 1828), pp. 75, l. 7–76, l. 10. Arab sources do make mention of such an artefact but tell a different story. According to Ibn al‐Athīr, during the Battle of the Straits Muslim soldiers captured a sword of Indian origin, whose inscription attributed it to Muḥammad, cf. Ibn al‐Athīr, *Chronicle*, *a*. 353, trans. Amari, *Biblioteca arabo‐sicula*, vol. 1, pp. 427–8.

^84^
Liutprand of Cremona, *Legatio*, c. 43, ed. P. Chiesa, *Liudprandi Cremonensis opera omnia*, CCCM 156 (Turnhout, 1998), p. 206, ll. 692–701.

^85^
Michael the Syrian XII.20, trans. Chabot, vol. 3, p. 100.

^86^
Al‐Ṭabarī, *History*, vol. 33, trans. Bosworth, pp. 116–17.

^87^
Kolia‐Dermitzaki, ‘Amorion’, pp. 141–2.

^88^
Y. Rotman, ‘Captif ou esclave? Entre marché d’esclaves et marché de captifs en Méditerranée médiévale’, in F. Guillén and S. Trabelsi (eds), *Les esclavage en Méditerranée* (Madrid, 2012), pp. 25–46.

^89^
Rotman, *Byzantine Slavery*.

^90^
Kaminiates, c. 78.1–3, ed. Böhlig, p. 67.

^91^

*Life of St Elias the Younger*, cc. 6 and 9, ed. G. Rossi Taibbi. *Vita di Sant’Elia il Giovane. Testo inedito con traduzione italiana* (Palermo, 1982), pp. 10 and 14.

^92^

*Life of St Cosmas the Hymnographer*, ed. Detorakes, pp. 269–74, ll. 133–317, esp. pp. 259–73, ll. 259–97.

^93^
Theophanes, ed. de Boor, p. 402, ll. 9–16.

^94^
Al‐Ṭabarī, *History*, vol. 33, trans. Bosworth, pp. 109–10.

^95^
Theophanes Continuatus VI.i.20, ed. Bekker, p. 366, ll. 11–17. On Leo, see also R. Khoury Odetallah, ‘Leo Tripolites – Ghulām Zurāfa and the Sack of Thessaloniki in 904’, *Byzantinoslavica* 56.1 (1995), pp. 97–102.

^96^
A. Guillou and W. Holtzman, ‘Zwei Katepansurkunden aus Tricarico’, *Quellen und Forschungen aus italienischen Archiven und Bibliotheken* 41 (1961), pp. 1–28 (= A. Guillou, *Studies on Byzantine Italy* (London, 1970), no. VII), at p. 18, ll. 1–9.

^97^
Cf. P. Sophoulis, *Byzantium and Bulgaria, 775*–*831* (Leiden and Boston, 2012).

^98^

*Scriptor Incertus*, ed. Iadevaia, p. 41, ll. 72–7.

^99^
V. Beševliev, *Die protobulgarischen Inschriften* (Berlin, 1963), pp. 220–1, ll. 15–30, no. 47.

^100^
Skylitzes, ed. Thurn, p. 330, ll. 2–5.

^101^
Skylitzes, ed. Thurn, pp. 341, ll. 13–22 and 342–3, ll. 52–67; Stephen of Tarōn III.33, trans. Greenwood, pp. 296–7.

^102^
Kaminiates, c. 78.8–9, ed. Böhlig, p. 67.

^103^
On this, see M. Campagnolo‐Pothitou, ‘Les échanges de prisonniers entre Byzance et l’Islam aux IXe et Xe siècles’, *Journal of Oriental and African Studies* 7 (1995), pp. 1–56; R.A. Khouri al Odetallah, *Άραβες και Βυζαντινοί. Το πρόβλημα των αιχμαλώτων πολέμου* (Thessaloniki, 1983); *idem*, ‘Ανεπίσημες ανταλλαγές’, pp. 109–13; A. Beihammer, ‘Strategies of Diplomacy and Ambassadors in Byzantine‐Muslim Relations on the Tenth and Eleventh Centuries’, in A. Becker and N. Drocourt (eds), *Ambassadeurs et ambassades au cœur des relations diplomatiques. Rome – Occident Médiéval – Byzance (VIIIe s. avant J.‐C. – XIIe s. après J.‐C.)* (Metz, 2012), pp. 371–400.

^104^
Bar Hebraeus, trans. Wallis Budge, p. 138.

^105^
Kaminiates, cc. 62.5–63.10, ed. Böhlig, pp. 53–4.

^106^
Kaminiates, c. 55.14, ed. Böhlig, p. 48; trans. Frendo and Fotiou, *Kaminiates*, p. 93.

^107^
Leo Choirosphaktes, *Letter* 23, ed. G. Kolias, *Léon Choerosphactès. Magistre, proconsul et patrice. Biographie – correspondance (Texte et traduction)* (Athens, 1939), p. 113, ll. 3–8 = *Letter* 18, ed. G. Strano, *Leone Choirosphaktes, corrispondenza. Introduzione, testo critico, traduzione e note di commento* (Catania, 2008), p. 72, ll. 3–8. For the date, see Campagnolo‐Pothitou, ‘Échanges’, p. 41.

^108^
Leo Choirosphaktes, *Letter* 15, ed. Kolias, p. 91 = *Letter* 21, ed. Strano, p. 86; Al‐Ṭabarī, *History*, vol. 38, trans. Rosenthal, p. 181; Bar Hebraeus, trans. Wallis Budge, pp. 154–5.

^109^
Peter of Sicily, *History of the Paulicians*, cc. 4 and 187–8, ed. C. Astruc *et al*., ‘Les sources grecques pour l’histoire des Pauliciens d’Asie Mineure’, *Travaux et Mémoires* 4 (1970), pp. 2–227, at pp. 3–67, esp. pp. 9, ll. 6–15 and 67, ll. 26–35.

^110^
N. Drocourt, ‘Religion and Diplomatic Peacemaking in the Middle Byzantine Period’, in Y. Friedman (ed.), *Religion and Peace. Historical Aspects* (Abingdon and New York, 2018), pp. 25–43.

^111^

*Kleinchroniken* 45.26, ed. P. Schreiner, *Die byzantinischen Kleinchroniken*, 3 vols, Corpus Fontium Historiae Byzantinae 12 (Vienna, 1975), vol. 1, p. 334; *Chronicle of Cambridge arab*., *a*. 6393, trans. Amari, vol. 1, p. 279; Ibn al‐Athīr, *Chronicle*, *a*. 271, trans. Amari, vol. 1, p. 399.

^112^
Rotman, *Byzantine Slavery*, pp. 36–9.

^113^
Al‐Ṭabarī, *History*, vol. 34, trans. J.L. Kraemer, pp. 42–3.

^114^
Kolia‐Dermitzaki, ‘Some Remarks’, pp. 614–20; Campagnolo‐Pothitou, ‘Les échanges’, pp. 19–22, 38 and 43–4.

^115^
Al‐Ṭabarī, *History*, vol. 34, trans. Kraemer, p. 39.

^116^
Bar Hebraeus, trans. Wallis Budge, p. 140.

^117^
Al‐Ṭabarī, *History*, vol. 34, trans. Kraemer, pp. 39–42; Bar Hebraeus, trans. Wallis Budge, p. 140. An exchange ratio of one to one (ψυχὶν ἀντὶ ψυχῖς, *sic*.) is attested also for negotiations with the Bulgars, see Beševliev, *Inschriften*, p. 190, ll. 12–15, no. 41.

^118^
Yaḥyā of Antioch, *History*, ed. Kratchkovsky and Vasiliev, pp. 772 and 774; Vasiliev, *Byzance et les Arabes*, vol. 2.2, pp. 125, 161–2 and 182–3.

^119^
Cf. M. Canard, ‘Quelques noms de personnages byzantins dans une pièce de poète Abû Firâs’, *Byzantion* 11 (1936), pp. 451–69 (= *idem*, *Byzance et les musulmans du Proche Orient* (London, 1973), no. IX).

^120^
F. Dölger, *Regesten der Kaiserurkunden des Oströmischen Reiches von 565*–*1453*, vol. 1.1.2: *Regesten von 867*–*1025*, rev. A.E. Müller and A. Beihammer, 2nd edn (Munich, 2003), pp. 125–6, no. 707d.

^121^
Yaḥyā of Antioch, *History*, ed. Kratchkovsky and Vasiliev, pp. 803–4.

^122^
Kaminiates, c. 66.4, ed. Böhlig, p. 56.

^123^

*Miracles of Saint Demetrios* V.284–8, ed. P. Lemerle, *Les plus anciens recueils des miracles de Saint Démétrius et la pénétration des slaves dans les Balkans*, 2 vols (Paris, 1979), vol. 1, p. 227, l. 16–p. 229, l. 3. For this incident, see M. Grigoriou‐Ioannidou, ‘The “καθ᾿ ἡμᾶς γλῶσσα” in the Mauros’ and Kouber’s Episode (*Miracula S. Demetrii* 291)’, in J. Burke and R. Scott (eds), *Byzantine Macedonia: Identity, Image and History. Papers from the Melbourne Conference, July 1995* (Leiden and Boston, 2000), pp. 89–101; D. Ziemann, *Vom Wandervolk zur Großmacht. Die Entstehung Bulgariens im frühen Mittelalter (7*.–*9. Jahrhundert)* (Cologne *et al*., 2007), pp. 135–41.

^124^
Symeon Logothete, *Chronicle* 131.9–12, ed. St. Wahlgren, *Symeonis Magistri et Logothetae chronicon*, Corpus Fontium Historiae Byzantinae 44 (Berlin and New York, 2006), pp. 235–7, ll. 70–109. On this episode, see I. Mladjov, ‘Trans‐Danubian Bulgaria: Reality and Fiction’, *Byzantine Studies*, ns 3 (1998), pp. 85–128; G. Simeonov, ‘The Region of the Danube Delta in the 7th–10th Centuries and the Case of the so‐called Lykostomion Maritime Province’, in J. Preiser‐Kapeller *et al*. (eds), *Proceedings of the Conference ‘Seasides of Byzantium. Harbours and Anchorages of a Mediterranean Empire’* (Mainz, 2021), pp. 233–54, esp. pp. 240–2.

^125^
Kolia‐Dermitzaki, ‘Amorion’, pp. 142–3; Patoura, *Aἰχμάλωτοι*, pp. 31–2.

^126^
Al‐Ṭabarī, History, vol. 33, trans. Bosworth, p. 116.

^127^
Kaminiates, c. 56.4–5, ed. Böhlig, p. 49.

^128^
Kaminiates, c. 76.4–5, ed. Böhlig, p. 65.

^129^
Theodosios the Monk, ed. Satta, p. 55, l. 29–p. 56, l. 8 (Az.) and p. 66, ll. 2–17 (Fi.); Kaminiates, c. 48, ed. Böhlig, pp. 42–3.

^130^
Kaminiates, c. 49.15–16, ed. Böhlig, p. 44.

^131^
Yaḥyā of Antioch, *History*, ed. Kratchkovsky and Vasiliev, p. 772.

^132^
Stephen of Tarōn III.10, trans. Greenwood, pp. 238–9; Yaḥyā of Antioch, *History*, ed. Kratchkovsky and Vasiliev, pp. 353–4.

^133^
Kaminiates, c. 55.2–6, ed. Böhlig, pp. 47–8.

^134^
B. Moulet, *Evêques, pouvoir et société à Byzance (VIIIe*–*XIe siècle). Territoires, communautés et individus dans la société provinciale byzantine* (Paris, 2011).

^135^
Michael the Syrian XII.20, trans. Chabot, vol. 3, p. 98; Bar Hebraeus, trans. Wallis Budge, p. 137.

^136^

*Chronicle of Cambridge arab*., *a*. 6434, trans. Amari, vol. 1, p. 283.

^137^
Arethas of Caesarea, *Scripta minora*, no. 36, ed. L.G. Westerink, *Arethae Archiepiscopi Caesariensis scripta minora*, 2 vols (Leipzig, 1968), vol. 1, pp. 285, ll. 2–7 and 287, ll. 13–18.

^138^
Arethas of Caesarea, *Scripta minora*, no. 36, ed. Westerink, vol. 1, p. 287, ll. 18–23.

^139^
John the Deacon, *Translation of St Severinus*, ed. Waitz, p. 453, ll. 6–10.

^140^
Al‐Ṭabarī, *History*, vol. 30, trans. Bosworth, p. 262.

^141^
Theodosios the Monk, ed. Satta, p. 55, l. 20–p. 56, l. 12, (Az.) and p. 65, l. 26–p. 66, l. 23 (Fi.).

^142^
Theodosios the Monk, ed. Satta, p. 55, l. 33–p. 56, l. 11, (Az.) and 66, ll. 1–21 (Fi.).

^143^
Theodosios the Monk, ed. Satta, pp. 57, ll. 30–6 (Az.) and 68, ll. 12–17 (Fi.).

^144^
Theophanes, ed. de Boor, p. 348, ll. 23–4.

^145^
Theophanes, ed. de Boor, p. 495, ll. 22–7.

^146^

*Synaxarium CP*, ed. H. Delehaye, *Synaxarium Ecclesiae Constantinopolitanae e Codice Sirmondiano nunc Berolinensi adiectis synaxariis selectis* (Brussels, 1902; repr. Louvain, 1954), p. 416, ll. 2–9.

^147^
U. Follieri and I. Dujčev, ‘Un acolutia inedita per i martiri di Bulgaria dell’anno 813’, *Byzantion* 33 (1963), pp. 71–106, at pp. 78, ll. 45–50 and 81, ll. 116–17.

^148^
L. Zanetti Domingues *et al*. (eds), *Women and Violence in the Late Medieval Mediterranean, ca. 1100*–*1500* (New York and Abingdon, 2021); Goldwyn, *Witness Literature*, pp. 183–95; N.M. El Cheikh, *Byzantium Viewed by the Arabs* (Cambridge, MA, 2004), pp. 123–38.

^149^
Michael the Syrian XII.20, trans. Chabot, vol. 3, p. 99; Bar Hebraeus, trans. Wallis Budge, p. 137.

^150^
Al‐Ṭabarī, *History*, vol. 33, trans. Bosworth, p. 117; Michael the Syrian XII.20, trans. Chabot, vol. 3, p. 100.

^151^
Kaminiates, cc. 60; 61.4–5 and 73.2–4, ed. Böhlig, pp. 52–3 and 62–3.

^152^

*Life of St Peter of Argos*, c. 15, ed. K. Kyriakopoulos, *Ἁγίου Πέτρου ἐπισκόπου Ἄργους βίος καὶ λόγοι. Εἰσαγωγή – κείμενον – μετάφρασις – σχόλια* (Athens, 1976), p. 244, ll. 238–42.

^153^
Kaminiates, c. 67.4–5, ed. Böhlig, pp. 56–7.

^154^
Brodman, ‘Captives or Prisoners’, p. 205.

^155^
Kaminiates, c. 73.10, ed. Böhlig, p. 63.

^156^

*Life of St Theoktiste of Lesbos*, c. 15, ed. H. Delehaye and P. Peeters, in *Acta Sanctorum*, *Novembris* IV (Brussels, 1925), p. 229 A–B.

^157^
On female mobility in Byzantine hagiography, see the contribution of Christodoulos Papavarnavas in this volume (with further bibliography).

^158^

*Un’ acolutia inedita*, ed. Follieri and Dujčev, pp. 79, ll. 57–62 and 81, ll. 116–22.

^159^
Theophanes Continuatus V.4, ed. Ševčenko, p. 20, ll. 9–11. See also Skylitzes, ed. Thurn, p. 116, l. 15; Genesios IV.24, ed. Lesmüller‐Werner and Thurn, p. 76, ll. 61–9.

^160^

*Book of Ceremonies* II.43, ed. G. Dagron *et al*., *Le livre des cérémonies*, 5 vols, Corpus Fontium Historiae Byzantinae 52 (Paris, 2020), vol. 3, p. 287, ll. 146–9.

^161^
The account of Basil’s childhood in Bulgarian captivity has provoked scholarly debate regarding its historicity: for the issue, see *PmbZ* # 832.

